# Supplementary material for: Salt Stress Tolerance in Casuarina glauca: Insights from the Branchlets Transcriptome
Source: Plants (Basel). 2022 Nov 1;11(21):2942. doi: 10.3390/plants11212942 (PMC9658546; doi:10.3390/plants11212942)
Supplement: Supplementary file 1 [file plants-11-02942-s001.zip › Table_S1.pdf]

Table S1. Sequencing data from *Casuarina glauca* nodulated by nitrogen-fixing *Frankia casuarinae* Thr (NOD$^+$) or non-nodulated plants supplied with KNO_3 (KNO_3^+), grown in three different salinity conditions (200, 400, and 600 mM NaCl), plus the control (0 mM NaCl). Raw reads, obtained after sequencing, generated trimmed reads after submission to quality filters with Trimmomatic software.

Plant-type	[NaCl] mM	Strand	Raw Reads			Trimmed Reads		
			Total Sequences	%GC	Min per base seq. quality	Total Sequences	%GC	Min per base seq. quality
NOD+	0	forward	37010538	46	28	26559076 (72%)	46	33
		reverse	37010538	46	15	26559076 (72%)	46	31
	200	forward	33417448	45	28	25496114 (76%)	45	33
		reverse	33417448	45	15	25496114 (76%)	45	31
	400	forward	33716971	45	28	22905738 (68%)	45	33
		reverse	33716971	45	15	22905738 (68%)	45	31
	600	forward	33881260	46	27	24012917 (71%)	45	33
		reverse	33881260	46	15	24012917 (71%)	45	31
$KNO_3^{\pm}$	0	forward	37089848	46	28	27361402 (74%)	45	33
		reverse	37089848	46	2	27361402 (74%)	46	31
	200	forward	38696059	46	28	27277745 (71%)	45	33
		reverse	38696059	46	15	27277745 (71%)	46	31
	400	forward	33546743	45	28	24471108 (73%)	45	33
		reverse	33546743	45	15	24471108 (73%)	45	31
	600	forward	35472156	46	28	26000474 (73%)	45	33
		reverse	35472156	46	15	26000474 (73%)	46	31
