# Supplementary material for: Salt Stress Tolerance in Casuarina glauca: Insights from the Branchlets Transcriptome
Source: Plants (Basel). 2022 Nov 1;11(21):2942. doi: 10.3390/plants11212942 (PMC9658546; doi:10.3390/plants11212942)
Supplement: Supplementary file 1 [file plants-11-02942-s001.zip › Table_S2.pdf]

Table S2. Quantification of basic quality metrics *de novo* transcriptome assembly of *Casuarina glauca*. Samples of *C. glauca* nodulated by nitrogen-fixing *Frankia casuarinae* Thr (NOD^+) and non-nodulated plants supplied with KNO_3 (KNO_3^+), grown at 0, 200, 400, and 600 mM NaCl, were combined to perform a *de novo* transcriptome assembly using Trinity software.

**Metrics**	**Values**
Total assembled contigs	181484
Smallest contig	183
Largest contig	17867
Total assembled bases	289780263
Total assembled genes	86202
Average contig length	1597
Median contig length	1036
# Contig sequences under 200	7
# Contig sequences over 1k	92205
# Contig sequences over 10k	170
# Contig sequences with ORF	76648
% Mean ORF	40
N90	727
N70	1904
N50	2792
N40	3260
N30	3805
N20	4520
N10	5729
% GC	41
