# Supplementary material for: Salt Stress Tolerance in Casuarina glauca: Insights from the Branchlets Transcriptome
Source: Plants (Basel). 2022 Nov 1;11(21):2942. doi: 10.3390/plants11212942 (PMC9658546; doi:10.3390/plants11212942)
Supplement: Supplementary file 1 [file plants-11-02942-s001.zip › Table_S3.pdf]

Table S3. Alignment statistics of *Casuarina glauca* nodulated by nitrogen-fixing *Frankia casuarinae* Thr (NOD^+) and non-nodulated plants supplied with KNO_3 (KNO_3^+) reads, from samples grown at 0, 200, 400, and 600 mM NaCl, against a *de novo* transcriptome assembly, performed with Trinity software using these same reads.

Statistics	Counts	Percentages
Total reads	204084574	-
Paired-end reads	204084574	100%
Uniquely aligned reads	30963937	15.2%
Reads aligned multiple times	165311208	81%
**Total aligned reads**	**196275145**	**96.2%**
Concordantly or discordantly unaligned reads	7374204	3.6%
**Concordantly unaligned and discordantly aligned reads**	**435225**	**0.2%**
Total concordantly unaligned reads	7809429	3.8%
Mates make up the pairs	14748408	-
Unaligned mates	3360270	22.8%[*]
Uniquely aligned mates	1263913	8.6%[*]
Mates aligned multiple times	10124225	68.7%[*]
**Total aligned mates**	**11388138**	**2.8%[**]**
**Overall alignment rate**		**99.2%**
