# Supplementary material for: Salt Stress Tolerance in Casuarina glauca: Insights from the Branchlets Transcriptome
Source: Plants (Basel). 2022 Nov 1;11(21):2942. doi: 10.3390/plants11212942 (PMC9658546; doi:10.3390/plants11212942)
Supplement: Supplementary file 1 [file plants-11-02942-s001.zip › Table_S4.pdf]

**Table S4.** Quality assessment of a *de novo* transcriptome assembly, using gVolante website with BUSCO v2.0.1 option. The transcriptome was performed with Trinity software, using *Casuarina glauca* nodulated by nitrogen-fixing *Frankia casuarinae* Thr ($NOD^+$) and non-nodulated plants supplied with $KNO_3$ ($KNO_3^+$) reads, from samples grown at 0, 200, 400, and 600 mM NaCl.

| **Completeness assessment** | **Values** |
|---|---|
| Total number of core genes queried | 1440 |
| Number of core genes detected | |
| **Complete** | **1366 (94.86%)** |
| Complete + Partial | 1412 (98.06%) |
| Number of missing core genes | 28 (1.94%) |
| Average number of orthologs per core genes | 1.92 |
| % of detected core genes that have more than 1 ortholog | 60.54 |
| Scores in BUSCO format | C:94.8%[S:37.4%,D:57.4%], F:3.2%,M:2.0% |

| **Length statistics and composition** | **Values** |
|---|---|
| Number of sequences | 181484 |
| Total length (nt) | 289780263 |
| Longest sequence (nt) | 17867 |
| Shortest sequence (nt) | 183 |
| Mean sequence length (nt) | 1597 |
| Median sequence length (nt) | 1036 |
| N50 sequence length (nt) | 2792 |
| L50 sequence count | 34704 |
| Number of sequences > 1K (nt) | 92205 (50.8%) |
| Number of sequences > 10K (nt) | 170 (0.1%) |
| Number of sequences > 100K (nt) | 0 |
| Number of sequences > 1M (nt) | 0 |
| Number of sequences > 10M (nt) | 0 |
| Base composition (%) | A: 30.98 |
| T: 27.94 | |
| G: 19.24 | |
| C: 21.85 | |
| N: 0.00 | |
| Other: 0.00 | |
| GC-content (%) | 41.09 |
| Number of non-ACGTN (nt) | 0 |
