# Supplementary material for: Salt Stress Tolerance in Casuarina glauca: Insights from the Branchlets Transcriptome
Source: Plants (Basel). 2022 Nov 1;11(21):2942. doi: 10.3390/plants11212942 (PMC9658546; doi:10.3390/plants11212942)
Supplement: Supplementary file 1 [file plants-11-02942-s001.zip › Table_S5.pdf]

Table S5. List of differentially expressed genes (DEGs) in both non-nodulated *Casuarina glauca* plants supplied with KNO_3 (KNO_3^+) and nodulated by nitrogen-fixing *Frankia casuarinae* Thr (NOD^+), grown in 400 mM NaCl relative to the control (0 mM NaCl). NCBI gene symbol and Protein Name, retrieved from NBCI blastx command line tool using UniprotKB/Swissprot database, and Log_2FC. DEGs were detected by DESeq, NOISeq and edgeR analysis with a False Discovery Rate < 0.05.

Gene Symbol	Protein Name	log_2FC	
		$KNO3^+$	NOD^+
**Upregulated**			
N/A	Unknown protein	2.64	2.62
SDI1	Protein SULFUR DEFICIENCY-INDUCED 1	3.00	3.46
HSP70	Heat shock 70 kDa protein	3.36	3.30
N/A	Unknown protein	3.58	2.96
N/A	Unknown protein	4.13	3.81
**Downregulated**			
RL5	Protein RADIALIS-like 5	-5.93	-7.79
YAH3	Histone H3.2	-5.48	-3.91
FLA12	Fasciclin-like arabinogalactan protein 12	-5.05	-4.54
CCD8	Carotenoid cleavage dioxygenase 8, chloroplastic	-5.03	-7.25
N/A	Unknown protein	-5.00	-6.33
PER64	Peroxidase 64	-4.89	-5.98
ROMT	Trans-resveratrol di-O-methyltransferase	-4.79	-3.73
GH3.2	Indole-3-acetic acid-amido synthetase GH3.2	-4.64	-3.41
N/A	Lectin	-4.60	-3.64

PME6	Probable pectinesterase/pectinesterase inhibitor 6	-4.22	-5.27
N/A	Unknown protein	-3.88	-3.43
N/A	Unknown protein	-3.84	-3.91
N/A	Unknown protein	-3.75	-3.39
LRK10L-1.4	LEAF RUST 10 DISEASE-RESISTANCE LOCUS RECEPTOR-LIKE PROTEIN KINASE-like 1.4	-3.70	-3.18
PBL9	Probable serine/threonine-protein kinase PBL9	-3.70	-2.77
FLS2	LRR receptor-like serine/threonine-protein kinase FLS2	-3.61	-3.63
N/A	Unknown protein	-3.53	-3.28
N/A	Unknown protein	-3.51	-4.12
N/A	Unknown protein	-3.42	-4.09
N/A	Unknown protein	-3.32	-3.91
SOT15	Cytosolic sulfotransferase 15	-3.21	-3.26
LECRK91	L-type lectin-domain containing receptor kinase IX.1	-3.20	-3.50
N/A	Unknown protein	-3.19	-2.87
N/A	Unknown protein	-3.06	-2.98
IRX15-L	Protein IRX15-LIKE	-3.03	-2.97
GAE6	UDP-glucuronate 4-epimerase 6	-2.65	-2.86
RL3	Protein RADIALIS-like 3	-2.62	-4.26
