# Supplementary material for: Salt Stress Tolerance in Casuarina glauca: Insights from the Branchlets Transcriptome
Source: Plants (Basel). 2022 Nov 1;11(21):2942. doi: 10.3390/plants11212942 (PMC9658546; doi:10.3390/plants11212942)
Supplement: Supplementary file 1 [file plants-11-02942-s001.zip › Table_S6.pdf]

Table S6. List of differentially expressed genes (DEGs) in both non-nodulated *Casuarina glauca* plants supplied with KNO_3 (KNO_3^+) and nodulated by nitrogen-fixing *Frankia casuarinae* Thr (NOD^+), grown in 600 mM NaCl relative to control (0 mM NaCl). NCBI gene symbol and Protein Name, retrieved from NBCI blastx command line tool using UniprotKB/Swissprot database, and Log_2FC. DEGs were detected by DESeq, NOISeq and edgeR analysis with a False Discovery Rate < 0.05.

Gene Symbol	Protein Name	log_2FC	
		$KNO3^+$	NOD^+
**Upregulated**			
N/A	Unknown protein	2.49	2.83
HSP70	Heat shock 70 kDa protein	3.12	2.73
SAT	Stemmadenine O-acetyltransferase	3.39	2.70
DTX56	Protein DETOXIFICATION 56	3.39	2.93
N/A	Unknown protein	3.44	2.20
N/A	Unknown protein	3.49	2.78
MAKR6	Probable membrane-associated kinase regulator 6	4.37	2.86
N/A	Unknown protein	4.52	3.70
N/A	Unknown protein	5.61	3.58
**Downregulated**			
N/A	Lectin	-6.60	-5.64
LAC22	Laccase-22	-6.46	-5.18
N/A	Unknown protein	-6.46	-6.24
N/A	Seed lectin	-6.39	-4.70
SERK4	Somatic embryogenesis receptor kinase 4	-5.95	-3.97
RL5	Protein RADIALIS-like 5	-5.93	-7.79

LECRK91	L-type lectin-domain containing receptor kinase IX.1	-5.78	-5.09
N/A	Unknown protein	-5.75	-4.39
LRK10L-1.4	LEAF RUST 10 DISEASE-RESISTANCE LOCUS RECEPTOR-LIKE PROTEIN KINASE-like 1.4	-5.70	-5.06
FLA12	Fasciclin-like arabinogalactan protein 12	-5.64	-3.86
PRB1	Pathogenesis-related protein 1	-5.60	-4.30
N/A	Unknown protein	-5.58	-5.09
WAKL9	Wall-associated receptor kinase-like 9	-5.52	-5.04
At2g40270	Inactive receptor-like serine/threonine-protein kinase At2g40270	-5.48	-3.58
IRX15-L	Protein IRX15-LIKE	-5.42	-3.46
CCD8	Carotenoid cleavage dioxygenase 8, chloroplastic	-5.39	-6.13
N/A	Unknown protein	-5.36	-2.93
N/A	Unknown protein	-5.36	-4.23
N/A	Unknown protein	-5.26	-6.33
N/A	Unknown protein	-5.21	-4.00
ROMT	Trans-resveratrol di-O-methyltransferase	-5.17	-4.83
N/A	Unknown protein	-5.13	-5.58
N/A	Unknown protein	-4.88	-5.74
N/A	Unknown protein	-4.83	-4.12
N/A	Unknown protein	-4.68	-4.71
N/A	Unknown protein	-4.62	-4.08
N/A	Unknown protein	-4.53	-4.30
N/A	Retrovirus-related Pol polyprotein from transposon TNT 1-94	-4.52	-4.46
N/A	Unknown protein	-4.49	-3.43
N/A	Unknown protein	-4.48	-3.95
GH3.2	Indole-3-acetic acid-amido synthetase GH3.2	-4.35	-3.05
PME6	Probable pectinesterase/pectinesterase inhibitor 6	-4.34	-4.66

N/A	Unknown protein	-4.28	-3.95
N/A	Unknown protein	-4.21	-3.54
N/A	Unknown protein	-4.09	-4.25
N/A	Unknown protein	-4.09	-3.75
TAO1	Disease resistance protein TAO1	-4.04	-5.73
N/A	Unknown protein	-4.02	-7.00
PLP2	Patatin-like protein 2	-3.94	-4.21
CRRSP38	Cysteine-rich repeat secretory protein 38	-3.94	-4.12
N/A	Unknown protein	-3.91	-2.88
ERF112	Ethylene-responsive transcription factor ERF112	-3.90	-4.66
PBL9	Probable serine/threonine-protein kinase PBL9	-3.87	-3.35
N/A	Unknown protein	-3.83	-4.16
CCR4	Serine/threonine-protein kinase-like protein CCR4	-3.80	-5.02
N/A	Unknown protein	-3.80	-4.39
N/A	Unknown protein	-3.76	-4.48
N/A	Unknown protein	-3.70	-3.91
N/A	Unknown protein	-3.69	-3.12
N/A	Unknown protein	-3.66	-3.29
SAG101	Senescence-associated carboxylesterase 101	-3.66	-3.88
At2g27310	F-box protein At2g27310	-3.64	-3.83
AATP1	AAA-ATPase ASD, mitochondrial	-3.63	-4.81
N/A	Unknown protein	-3.58	-4.17
N/A	Unknown protein	-3.58	-4.59
N/A	Unknown protein	-3.56	-2.89
N/A	Unknown protein	-3.52	-4.98
N/A	Unknown protein	-3.52	-3.55

AOS1	Allene oxide synthase 1, chloroplastic	-3.52	-3.77
N/A	Unknown protein	-3.49	-3.00
RL3	Protein RADIALIS-like 3	-3.49	-3.40
N/A	Unknown protein	-3.48	-3.55
SOBIR1	Leucine-rich repeat receptor-like serine/threonine/tyrosine-protein kinase SOBIR1	-3.37	-4.44
SOT15	Cytosolic sulfotransferase 15	-3.36	-4.65
N/A	Unknown protein	-3.32	-3.25
DREB1D	Dehydration-responsive element-binding protein 1D	-3.31	-4.73
N/A	Unknown protein	-3.30	-3.92
N/A	Unknown protein	-3.26	-3.87
RGA5	Disease resistance protein RGA5	-3.16	-4.08
N/A	Unknown protein	-3.16	-2.87
CXE6	Probable carboxylesterase 6	-3.14	-4.14
NAC090	NAC domain-containing protein 90	-3.14	-3.49
CYP74A	Allene oxide synthase, chloroplastic	-3.13	-3.53
N/A	Unknown protein	-3.12	-3.19
N/A	Unknown protein	-3.12	-3.66
WRKY70	Probable WRKY transcription factor 70	-3.12	-3.26
N/A	Unknown protein	-3.10	-5.29
EXO70H1	Exocyst complex component EXO70H1	-3.10	-3.81
N/A	Unknown protein	-3.07	-3.30
N/A	Unknown protein	-3.07	-2.98
N/A	Unknown protein	-3.06	-2.95
LRK10	Rust resistance kinase Lr10	-3.05	-3.26
N/A	Unknown protein	-3.05	-3.19
UGT76E2	UDP-glycosyltransferase 76E2	-3.05	-2.80

PHT1-12	Probable inorganic phosphate transporter 1-12	-3.04	-3.58
N/A	Unknown protein	-3.03	-3.29
PUMP5	Mitochondrial uncoupling protein 5	-2.96	-3.00
CML37	Calcium-binding protein CML37	-2.95	-3.60
pod	Peroxidase 15	-2.93	-5.53
N/A	Unknown protein	-2.89	-2.84
N/A	Unknown protein	-2.88	-3.22
N/A	Unknown protein	-2.86	-3.33
N/A	Unknown protein	-2.78	-4.02
PUB17	U-box domain-containing protein 17	-2.76	-3.23
N/A	Unknown protein	-2.71	-5.25
EDS1	Protein EDS1L	-2.70	-3.43
N/A	Cytochrome P450 CYP736A12	-2.67	-3.46
N/A	Unknown protein	-2.65	-4.39
N/A	Unknown protein	-2.64	-4.26
At4g03230	G-type lectin S-receptor-like serine/threonine-protein kinase At4g03230	-2.61	-3.07
SAP12	Zinc finger AN1 domain-containing stress-associated protein 12	-2.60	-2.89
N/A	Unknown protein	-2.58	-2.86
N/A	Unknown protein	-2.58	-3.84
PK1	Putative receptor protein kinase ZmPK1	-2.58	-3.59
N/A	Unknown protein	-2.57	-3.64
N/A	Unknown protein	-2.57	-4.03
N/A	Unknown protein	-2.57	-3.55
RPP13L4	Disease resistance RPP13-like protein 4	-2.56	-3.31
N/A	Unknown protein	-2.55	-2.82
UGT85A23	7-deoxyloganetin glucosyltransferase	-2.54	-3.74

N/A	Unknown protein	-2.50	-3.22
GNS1	Glucan endo-1,3-beta-glucosidase, basic isoform	-2.50	-3.32
N/A	Unknown protein	-2.49	-3.13
N/A	Unknown protein	-2.49	-3.58
N/A	Unknown protein	-2.44	-4.23
DTX49	Protein DETOXIFICATION 49	-2.41	-4.75
At5g24080	G-type lectin S-receptor-like serine/threonine-protein kinase At5g24080	-2.40	-3.93
N/A	Unknown protein	-2.37	-3.19
N/A	Unknown protein	-2.36	-3.35
At2g30020	Probable protein phosphatase 2C 25	-2.34	-3.98
N/A	Unknown protein	-2.31	-2.90
DAR4	Protein DA1-related 4	-2.30	-3.35
PUB19	U-box domain-containing protein 19	-2.28	-3.03
N/A	Unknown protein	-2.24	-2.89
LECRK3	G-type lectin S-receptor-like serine/threonine-protein kinase LECRK3	-2.22	-3.50
CRT3	Calreticulin-3	-2.22	-2.94
