# Supplementary material for: Salt Stress Tolerance in Casuarina glauca: Insights from the Branchlets Transcriptome
Source: Plants (Basel). 2022 Nov 1;11(21):2942. doi: 10.3390/plants11212942 (PMC9658546; doi:10.3390/plants11212942)
Supplement: Supplementary file 1 [file plants-11-02942-s001.zip › Table_S7.pdf]

Table S7. Full list of significantly differentially expressed genes (DEGs) by non-nodulated *Casuarina glauca* plants supplied with KNO_3 (KNO_3^+), grown in 200, 400, and 600 mM NaCl. NCBI gene symbol, protein ID, Protein Name, E-value and Identities retrieved from NBCI blastx command line tool, using UniprotKB/Swissprot database, and Log2FC (positive values in bold). DEGs refers to each salinity-treatment in comparison with the control NaCl, respectively, 200: 200 *vs*. 0 mM NaCl, 400: 400 *vs*. 0 mM NaCl and 600: 600 *vs*. 0 mM NaCl), detected by DESeq, NOISeq and edgeR analysis with a False Discovery Rate < 0.05.

Gene Symbol	Protein ID	Protein Name	E-value	Identities	Log2FC		
					200	400	600
LRK10L-1.4	LRL14_ARATH	LEAF RUST 10 DISEASE-RESISTANCE LOCUS RECEPTOR-LIKE PROTEIN KINASE-like 1.4	5E-07	45	-	-3.700	-5.700
SAUR20	SAU20_ARATH	Auxin-responsive protein SAUR20	3E-23	65	-	-	-3.459
FLS2	FLS2_ARATH	LRR receptor-like serine/threonine-protein kinase FLS2	2E-63	56	-	-3.609	-
LRK10	LRK10_WHEAT	Rust resistance kinase Lr10	8E-64	56	-	-	-3.052
WLIM1	WLIM1_ARATH	LIM domain-containing protein WLIM1	1E-29	76	-	-2.682	-3.874
KIN12E	KN12E_ARATH	Kinesin-like protein KIN-12E	1E-32	43	-	-	-3.459
CCR3	ACCR3_ARATH	Putative serine/threonine-protein kinase-like protein CCR3	0	56	-	-3.204	-3.237
DSC1	DSC1_ARATH	Disease resistance-like protein DSC1	3E-15	46	-	-	-2.489
CSLD3	CSLD3_ARATH	Cellulose synthase-like protein D3	0	89	-	-2.694	-
PUB19	PUB19_ARATH	U-box domain-containing protein 19	9E-10	43	-	-	-2.284
CRK27	CRK27_ARATH	Cysteine-rich receptor-like protein kinase 27	6E-17	49	-	-	-4.907
N/A	PME_DAUCA	Pectinesterase	5E-33	88	-	-3.760	-
GALT6	B3GTJ_ARATH	Hydroxyproline O-galactosyltransferase GALT6	2E-07	70	-	-3.087	-2.628
ZED1	ZED1_ARATH	Non-functional pseudokinase ZED1	9E-04	47	-	-	-2.615
SAG101	SG101_ARATH	Senescence-associated carboxylesterase 101	8E-27	51	-	-	-3.659
N	TMVRN_NICGU	TMV resistance protein N	3E-04	84	-	-	-3.459

LECRK111	LK111_ARATH	Putative L-type lectin-domain containing receptor kinase I.11	6E-124	51	-	**2.369**	-
TUBB3	TBB3_ANEPH	Tubulin beta-3 chain	2E-43	97	-	-3.503	-2.766
AT7	AT7_ORYSJ	Acyl transferase 7	8E-52	41	-	-	-3.016
ACA13	ACA13_ARATH	Putative calcium-transporting ATPase 13, plasma membrane-type	5E-43	78	-	-6.476	-5.476
BGAL3	BGAL3_ARATH	Beta-galactosidase 3	9E-38	56	-	-3.202	-
RLP1	RLP1_ARATH	Receptor-like protein 1	1E-26	47	-	-	-2.907
N/A	LEC_ONOVI	Lectin	4E-08	57	-	-4.600	-6.600
WRKY19	WRK19_ARATH	Probable WRKY transcription factor 19	3E-05	50	-	-	-3.170
SAT	SAT_CATRO	Stemmadenine O-acetyltransferase	2E-16	47	-	-	**3.392**
DTX56	DTX56_ARATH	Protein DETOXIFICATION 56	1E-144	63	-	-	**3.392**
PK1	KPRO_MAIZE	Putative receptor protein kinase ZmPK1	2E-80	59	-	-	-2.575
CYP82A1	C82A1_PEA	Cytochrome P450 82A1	9E-40	62	-	-	-2.678
N/A	TBA_BLEJA	Tubulin alpha chain	5E-06	100	-	-2.871	-2.519
GAUT12	GAUTC_ARATH	Probable galacturonosyltransferase 12	0	77	-	-3.662	-4.790
CCD8	CCD8_ARATH	Carotenoid cleavage dioxygenase 8, chloroplastic	4E-05	40	-	-5.027	-5.390
DTX49	DTX49_ARATH	Protein DETOXIFICATION 49	2E-24	69	-	-	-2.406
DAR4	DAR4_ARATH	Protein DA1-related 4	3E-10	47	-	-	-2.298
PLR_Fi1	PILR1_FORIN	Bifunctional pinoresinol-lariciresinol reductase	2E-59	86	-	-	-2.716
EDS1	EDS1C_ARATH	Protein EDS1	4E-14	56	-	-4.392	-4.392
N/A	CHIT2_TULSB	Chitinase 2	5E-46	73	-	-	**2.807**
CNGC4	CNGC4_ARATH	Cyclic nucleotide-gated ion channel 4	4E-32	90	-	-	**2.672**
At3g56050	Y3565_ARATH	Probable inactive receptor-like protein kinase At3g56050	1E-46	48	-	-2.861	-4.649
SBT1.8	SBT18_ARATH	Subtilisin-like protease SBT1.8	6E-56	85	-	-3.087	-
RPP13L4	R13L4_ARATH	Disease resistance RPP13-like protein 4	5E-35	60	-	-	-2.559
PEX1	PLRX1_ARATH	Pollen-specific leucine-rich repeat extensin-like protein 1	2E-45	61	-	-4.087	-
N/A	RLA0_LUPLU	60S acidic ribosomal protein P0	2E-37	79	-	-	**2.627**

pod	PER15_IPOBA	Peroxidase 15	6E-65	64	-	-	-2.934
PAL	PALY_PINTA	Phenylalanine ammonia-lyase	4E-119	70	-	-	-3.700
NDA1	NDA1_ARATH	Internal alternative NAD(P)H-ubiquinone oxidoreductase A1, mitochondrial	2E-20	80	-	-3.688	-4.858
RBOHD	RBOHD_ARATH	Respiratory burst oxidase homolog protein D	8E-39	87	-	-2.784	-
MIK2	MIK2_ARATH	MDIS1-interacting receptor like kinase 2	1E-17	53	-	-3.059	-3.059
PDI	PDI_DATGL	Protein disulfide-isomerase	2E-06	68	-	-	-2.585
At5g42100	E1310_ARATH	Glucan endo-1,3-beta-glucosidase 10	2E-20	47	-	-2.980	-2.565
At4g10390	Y4103_ARATH	Probable receptor-like protein kinase At4g10390	4E-39	61	-	-3.585	-
LAC14	LAC14_ARATH	Laccase-14	6E-38	50	-	-3.571	-3.152
WAKL9	WAKLH_ARATH	Wall-associated receptor kinase-like 9	1E-06	49	-	-3.202	-5.524
FLA10	FLA10_ARATH	Fasciclin-like arabinogalactan protein 10	2E-36	83	-	-3.921	-
PGL3	PGL3_ARATH	Polygalacturonase 1 beta-like protein 3	3E-60	64	-	-5.073	-3.199
DOT4	PP320_ARATH	Pentatricopeptide repeat-containing protein DOT4, chloroplastic	0	63	-	-3.509	-5.624
FER	FERON_ARATH	Receptor-like protein kinase FERONIA	1E-31	47	-	-	-3.000
CSLA9	CSLA9_ARATH	Glucomannan 4-beta-mannosyltransferase 9	2E-31	78	-	-4.248	-3.248
At3g10080	GL32_ARATH	Germin-like protein subfamily 3 member 2	7E-31	65	-	-6.658	-5.073
At3g61750	B561I_ARATH	Cytochrome b561 and DOMON domain-containing protein At3g61750	1E-50	60	-	-3.605	-6.775
PLT6	PLT6_ARATH	Probable polyol transporter 6	4E-75	67	-	-	**4.555**
At5g24080	Y5248_ARATH	G-type lectin S-receptor-like serine/threonine-protein kinase At5g24080	9E-27	57	-	-	-2.397
AOS1	AOS1_SOLLC	Allene oxide synthase 1, chloroplastic	3E-113	67	-	-2.753	-3.519
TOGT1	SCGT_TOBAC	Scopoletin glucosyltransferase	2E-34	66	-	**2.290**	**2.713**
PME3	PME3_ARATH	Pectinesterase/pectinesterase inhibitor 3	9E-56	78	-	-3.539	-
N/A	21KD_DAUCA	21 kDa protein	5E-47	51	-	-4.115	-3.700
PBL9	PBL9_ARATH	Probable serine/threonine-protein kinase PBL9	9E-08	81	-	-3.700	-3.870
At2g27310	FB119_ARATH	F-box protein At2g27310	4E-56	54	-	-3.799	-3.642

FLA17	FLA17_ARATH	Fasciclin-like arabinogalactan protein 17	1E-175	76	-	-	-3.043
At5g66900	DRL42_ARATH	Probable disease resistance protein At5g66900	2E-11	50	-	-2.459	-
PME6	PME6_ARATH	Probable pectinesterase/pectinesterase inhibitor 6	3E-146	68	-	-4.220	-4.341
At4g32285	CAP1_ARATH	Probable clathrin assembly protein At4g32285	2E-25	58	-	-2.652	-
CESA9	CESA9_ORYSI	Cellulose synthase A catalytic subunit 9 [UDP-forming]	7E-56	72	-	-3.524	-6.109
MAKR6	MAKR6_ARATH	Probable membrane-associated kinase regulator 6	2E-07	78	-	-	**4.366**
TAO1	TAO1_ARATH	Disease resistance protein TAO1	6E-10	41	-	-2.722	-4.044
AMT1-1	AMT11_ARATH	Ammonium transporter 1 member 1	0	82	-	-	-2.417
SOBIR1	SBIR1_ARATH	Leucine-rich repeat receptor-like serine/threonine/tyrosine-protein kinase SOBIR1	7E-124	80	-	-2.614	-3.373
RCOM_0699480	Y232_RICCO	Glycosyltransferase family 92 protein RCOM_0530710	3E-102	49	-	-2.701	-2.785
HSP70	HSP70_SOYBN	Heat shock 70 kDa protein	3E-70	84	-	**3.363**	**3.123**
GH3.2	GH32_ARATH	Indole-3-acetic acid-amido synthetase GH3.2	2E-118	74	-	-4.644	-4.354
PATL4	PATL4_ARATH	Patellin-4	1E-08	78	-	-5.129	-
OFUT10	OFT10_ARATH	O-fucosyltransferase 10	3E-05	64	-	-	-3.700
GALS1	GALS1_ARATH	Galactan beta-1,4-galactosyltransferase GALS1	8E-30	75	-	-3.115	-
At1g33260	Y1332_ARATH	Probable receptor-like protein kinase At1g33260	2E-06	57	-	-4.170	-4.170
LRX4	LRX4_ARATH	Leucine-rich repeat extensin-like protein 4	2E-155	79	-	-3.781	-
WRKY70	WRK70_ARATH	Probable WRKY transcription factor 70	1E-29	66	-	-2.539	-3.116
PNC1	PER1_ARAHY	Cationic peroxidase 1	7E-39	77	-	-	**2.330**
N/A	BCP_PEA	Blue copper protein	3E-33	79	-	-	-2.874
TUBB1	TBB1_MAIZE	Tubulin beta-1 chain	9E-39	95	-	-3.059	-
CYP81E8	C81E8_MEDTR	Cytochrome P450 81E8	1E-04	83	-	-4.346	-3.346
At4g22030	FB333_ARATH	Probable F-box protein At4g22030	6E-14	64	-	-	-4.248
P85	SDEH4_ARATH	Senescence/dehydration-associated protein At4g35985, chloroplastic	6E-14	57	-	-2.415	-
N/A	PME_PRUPE	Pectinesterase/pectinesterase inhibitor PPE8B	3E-11	66	-	-4.392	-

PGDH2	SERA2_ARATH	D-3-phosphoglycerate dehydrogenase 2, chloroplastic	3E-41	86	-	-3.558	-3.406
FLA12	FLA12_ARATH	Fasciclin-like arabinogalactan protein 12	5E-14	72	-	-5.055	-5.640
CESA7	CESA7_ARATH	Cellulose synthase A catalytic subunit 7 [UDP-forming]	5E-75	87	-	-4.420	-4.420
EXO70H1	E70H1_ARATH	Exocyst complex component EXO70H1	1E-24	54	-	-	-3.102
XCP1	XCP1_ARATH	Cysteine protease XCP1	1E-22	64	-	-6.883	-6.883
PUMP5	PUMP5_ARATH	Mitochondrial uncoupling protein 5	2E-24	77	-	-2.707	-2.964
ERF020	ERF20_ARATH	Ethylene-responsive transcription factor ERF020	2E-21	79	-4.059	-5.644	-3.644
At3g27390	Y3739_ARATH	Uncharacterized membrane protein At3g27390	4E-74	71	-	-	**4.881**
NHL10	NHL10_ARATH	NDR1/HIN1-like protein 10	5E-17	48	-	-	-2.377
UGT709C2	UGT8_CATRO	7-deoxyloganetic acid glucosyltransferase	4E-50	72	-	-	**3.415**
LYK4	LYK4_ARATH	LysM domain receptor-like kinase 4	5E-12	45	-	-	-4.170
ROMT	ROMT_VITVI	Trans-resveratrol di-O-methyltransferase	1E-59	50	-	-4.787	-5.166
ACA12	ACA12_ARATH	Calcium-transporting ATPase 12, plasma membrane-type	5E-69	67	-	-3.087	-4.087
CRT3	CALR3_ARATH	Calreticulin-3	2E-29	79	-	-	-2.220
CYP707A7	ABAH3_ORYSI	Abscisic acid 8'-hydroxylase 3	1E-13	69	-	-	**2.303**
LRX6	LRX6_ARATH	Leucine-rich repeat extensin-like protein 6	1E-160	68	-	-3.185	-4.408
SDI1	SDI1_ARATH	Protein SULFUR DEFICIENCY-INDUCED 1	3E-08	56	-	**3.000**	-
LECRK3	LERK3_ORYSI	G-type lectin S-receptor-like serine/threonine-protein kinase LECRK3	4E-72	56	-	-	-2.225
GH3.1	GH31_ARATH	Probable indole-3-acetic acid-amido synthetase GH3.1	3E-62	83	-	-4.883	-4.298
ASPG2	ASPG2_ARATH	Protein ASPARTIC PROTEASE IN GUARD CELL 2	3E-53	63	-	-3.369	-3.954
At4g37220	CRPM4_ARATH	Cold-regulated 413 plasma membrane protein 4	8E-07	42	-	-3.719	-
At2g40270	Y2027_ARATH	Inactive receptor-like serine/threonine-protein kinase At2g40270	8E-06	62	-	-3.306	-5.476
PER64	PER64_ARATH	Peroxidase 64	5E-59	60	-	-4.891	-4.476
PUB33	PUB33_ARATH	U-box domain-containing protein 33	5E-13	60	-	-	**2.436**
UGT76D1	U76D1_ARATH	UDP-glycosyltransferase 76D1	1E-05	63	-	-2.526	-3.848
COBL4	COBL4_ARATH	COBRA-like protein 4	3E-21	87	-	-	-3.700

At5g39865	Y5986_ARATH	Uncharacterized protein At5g39865	9E-08	71	-	-2.821	-2.336
EDS1	EDS1L_ARATH	Protein EDS1L	2E-37	45	-	-	-2.704
CXE17	CXE17_ARATH	Probable carboxylesterase 17	4E-19	49	-	-	-3.700
GLR2.9	GLR29_ARATH	Glutamate receptor 2.9	2E-06	43	-	-	-2.858
UGT76E2	U76E2_ARATH	UDP-glycosyltransferase 76E2	8E-56	70	-	-2.844	-3.048
YAH3	H32_LILLO	Histone H3.2	9E-42	99	-	-5.476	-3.668
At2g17340	D89S2_ARATH	Damage-control phosphatase At2g17340	2E-160	86	-	-	-4.536
CYP74A	CP74A_ARATH	Allene oxide synthase, chloroplastic	2E-40	78	-	-2.421	-3.134
LRK10L-1.5	LRL15_ARATH	LEAF RUST 10 DISEASE-RESISTANCE LOCUS RECEPTOR-LIKE PROTEIN KINASE-like 1.5	0	63	-	-3.568	-
DTX27	DTX27_ARATH	Protein DETOXIFICATION 27	3E-24	60	-	-	**2.690**
At4g03230	Y4230_ARATH	G-type lectin S-receptor-like serine/threonine-protein kinase At4g03230	1E-46	70	-	-	-2.615
AT9	AT9_ORYSJ	Acyl transferase 9	2E-17	44	-	-	-3.858
STN8	STN8_ARATH	Serine/threonine-protein kinase STN8, chloroplastic	0	69	-	-4.212	-5.119
TAF6	TAF6_ARATH	Transcription initiation factor TFIID subunit 6	2E-121	79	-	-4.827	-5.505
CCR4	ACCR4_ARATH	Serine/threonine-protein kinase-like protein CCR4	3E-83	40	-	-2.797	-3.803
SAP12	SAP12_ARATH	Zinc finger AN1 domain-containing stress-associated protein 12	2E-26	58	-	-3.793	-2.600
Bp10	ASOL_BRANA	L-ascorbate oxidase homolog	5E-31	57	-	-4.170	-
RLP15	RLP15_ARATH	Receptor-like protein 15	3E-19	44	-	-3.020	-
LAC9	LAC9_ORYSJ	Putative laccase-9	1E-34	71	-	-4.346	-4.931
SCPL4	SCP4_ARATH	Serine carboxypeptidase-like 4	3E-15	53	-	-	-3.807
NAC090	NAC90_ARATH	NAC domain-containing protein 90	2E-43	67	-	-	-3.141
RE1	POLR1_ARATH	Retrovirus-related Pol polyprotein from transposon RE1	3E-72	42	-	-2.588	-3.588
RDR1	RDR1_ARATH	RNA-dependent RNA polymerase 1	8E-32	60	-	-2.585	-2.826
NSP5	NSP5_ARATH	Nitrile-specifier protein 5	3E-27	59	-	-	**2.487**
N/A	PRU1_PRUAR	Major allergen Pru ar 1	9E-39	45	-	-4.033	-5.448

CRRSP38	CRR38_ARATH	Cysteine-rich repeat secretory protein 38	2E-08	44	-	-2.939	-3.939
LRX2	LRX2_ARATH	Leucine-rich repeat extensin-like protein 2	0	75	-	-5.157	-
ERF112	EF112_ARATH	Ethylene-responsive transcription factor ERF112	2E-17	83	-	-3.462	-3.898
BHLH92	BH092_ARATH	Transcription factor bHLH92	1E-14	54	-	-	-3.322
PER53	PER53_ARATH	Peroxidase 53	1E-18	46	-	-3.036	-
N/A	POLX_TOBAC	Retrovirus-related Pol polyprotein from transposon TNT 1-94	1E-36	49	-	-	-4.524
SOT15	SOT15_ARATH	Cytosolic sulfotransferase 15	5E-141	61	-	-3.210	-3.364
XXT3	XXT3_ARATH	Probable xyloglucan 6-xylosyltransferase 3	2E-61	86	-	-3.063	-
N/A	PME1_FICPW	Pectinesterase/pectinesterase inhibitor	6E-15	51	-	-2.921	-
RGA5	RGA5R_ORYSJ	Disease resistance protein RGA5	3E-04	44	-	-	-3.160
RFS6	RFS6_ARATH	Probable galactinol--sucrose galactosyltransferase 6	6E-33	73	-	-3.000	-2.415
CML41	CML41_ARATH	Probable calcium-binding protein CML41	1E-49	50	-	-2.814	-3.213
DREB1D	DRE1D_ARATH	Dehydration-responsive element-binding protein 1D	2E-32	71	-	-4.044	-3.307
At2g30020	P2C25_ARATH	Probable protein phosphatase 2C 25	8E-32	74	-	-	-2.344
CSLD2	CSLD2_ARATH	Cellulose synthase-like protein D2	3E-31	73	-	-3.585	-
At5g22810	GDL78_ARATH	GDSL esterase/lipase At5g22810	2E-27	73	-	-3.248	-
ARF16	ARFP_ARATH	Auxin response factor 16	2E-30	72	-	**2.644**	-
GT-3B	TGT3B_ARATH	Trihelix transcription factor GT-3b	1E-04	55	-3.807	-3.807	-
WAXY	SSG1_MANES	Granule-bound starch synthase 1, chloroplastic/amyloplastic	6E-04	76	-	**3.459**	-
At1g18250	TLPH_ARATH	Thaumatin-like protein	6E-66	73	-	-3.759	-2.372
MUR1	GMD2_ARATH	GDP-mannose 4,6 dehydratase 2	7E-15	68	-	-	-2.541
HMA1	HMA1_ARATH	Probable cadmium/zinc-transporting ATPase HMA1, chloroplastic	1E-69	72	-	-4.585	-
At1g32860	E1311_ARATH	Glucan endo-1,3-beta-glucosidase 11	3E-47	45	-	-3.202	-
N/A	MYRS_QUEIL	Myrcene synthase, chloroplastic	4E-22	65	-	**3.129**	-
PRB1	PRB1_ARATH	Pathogenesis-related protein 1	6E-66	62	-	-	-5.600
PUB17	PUB17_ARATH	U-box domain-containing protein 17	7E-35	70	-	-2.758	-2.758

RPP1	RPP1_ARATH	Probable disease resistance protein RPP1	1E-04	56	-	-	-4.322
RL5	RADL5_ARATH	Protein RADIALIS-like 5	2E-28	70	-	-5.931	-5.931
CHS2	CHS2_CAMSI	Chalcone synthase 2	7E-47	96	-	-	-2.305
GNS1	E13B_PRUPE	Glucan endo-1,3-beta-glucosidase, basic isoform	1E-157	68	-	-	-2.501
RAP2-3	RAP23_ARATH	Ethylene-responsive transcription factor RAP2-3	4E-19	62	-	-	**2.353**
N/A	C7A12_PANGI	Cytochrome P450 CYP736A12	1E-70	63	-	-	-2.665
RGA1	RGA1_SOLBU	Putative disease resistance protein RGA1	2E-04	41	-	-	-3.209
CXE6	CXE6_ARATH	Probable carboxylesterase 6	8E-14	40	-	-	-3.143
UGT87A1	U87A1_ARATH	UDP-glycosyltransferase 87A1	9E-85	46	-	**2.824**	-
RL3	RADL3_ARATH	Protein RADIALIS-like 3	1E-28	75	-	-2.623	-3.490
rpoC1	RPOC1_CHLSC	DNA-directed RNA polymerase subunit beta'	5E-25	81	-	-	**2.468**
N/A	BABL_CUCSA	Basic blue protein	3E-25	65	-	-	-2.950
GATL2	GATL2_ARATH	Probable galacturonosyltransferase-like 2	2E-22	56	-	-4.087	-
TIR	TIR_ARATH	Toll/interleukin-1 receptor-like protein	3E-32	51	-	-2.893	-2.700
At1g12500	PT112_ARATH	Probable sugar phosphate/phosphate translocator At1g12500	1E-78	87	-	-	-3.492
GAE6	GAE6_ARATH	UDP-glucuronate 4-epimerase 6	6E-97	93	-	-2.647	-
BXL7	BXL7_ARATH	Probable beta-D-xylosidase 7	9E-108	71	-	-3.794	-
KIN14Q	KN14Q_ARATH	Kinesin-like protein KIN-14Q	3E-18	56	-	-4.030	-4.030
CYCD1-1	CCD11_ARATH	Cyclin-D1-1	6E-17	61	-	-	-2.536
PLP2	PLP2_ARATH	Patatin-like protein 2	1E-44	66	-	-	-3.939
LAC22	LAC22_ORYSJ	Laccase-22	4E-138	64	-	-4.344	-6.459
ROPGAP2	RGAP2_ARATH	Rho GTPase-activating protein 2	1E-20	65	-	-4.644	-
At1g73050	MDLL_ARATH	(R)-mandelonitrile lyase-like	2E-24	58	-	-	-2.300
TBL30	TBL30_ARATH	Protein trichome birefringence-like 30	1E-05	86	-	-	-3.059
AATP1	ASD_ARATH	AAA-ATPase ASD, mitochondrial	3E-15	50	-	-	-3.629
LECRK91	LRK91_ARATH	L-type lectin-domain containing receptor kinase IX.1	4E-33	68	-	-3.196	-5.781

GT17	GT17_ARATH	Probable xyloglucan galactosyltransferase GT17	7E-15	57	-	-3.617	-
IRX15-L	IX15L_ARATH	Protein IRX15-LIKE	3E-25	61	-	-3.026	-5.418
APG	APG2_ARATH	GDSL esterase/lipase APG	8E-19	65	-	-	-3.907
At5g20050	Y5005_ARATH	Probable receptor-like protein kinase At5g20050	3E-145	64	-	-	-2.554
FLZ17	FLZ17_ARATH	FCS-Like Zinc finger 17	7E-06	60	-	-	-2.648
CML37	CML37_ARATH	Calcium-binding protein CML37	1E-19	50	-	-	-2.948
SERK4	SERK4_ARATH	Somatic embryogenesis receptor kinase 4	1E-05	40	-	-4.369	-5.954
PLP1	PLP1_ARATH	Patatin-like protein 1	3E-26	88	-	-	-3.607
TAR1	TAR1_YEAST	Protein TAR1	2E-06	78	-	-4.248	-
N/A	LECS_VATMA	Seed lectin	2E-16	40	-	-3.807	-6.392
UGT85A23	UGT6_CATRO	7-deoxyloganetin glucosyltransferase	2E-142	55	-	-	-2.542
ABCB13	AB13B_ARATH	ABC transporter B family member 13	3E-59	75	-	-3.907	-
PHT1-12	PT112_ORYSJ	Probable inorganic phosphate transporter 1-12	2E-32	79	-	-2.596	-3.043
