# Supplementary material for: Salt Stress Tolerance in Casuarina glauca: Insights from the Branchlets Transcriptome
Source: Plants (Basel). 2022 Nov 1;11(21):2942. doi: 10.3390/plants11212942 (PMC9658546; doi:10.3390/plants11212942)
Supplement: Supplementary file 1 [file plants-11-02942-s001.zip › Table_S8.pdf]

Table S8. Full list of significantly differentially expressed genes (DEGs) in branchlets of *Casuarina glauca* nodulated by nitrogen-fixing *Frankia casuarinae* Thr (NOD^+), grown in 200, 400, and 600 mM NaCl. NCBI gene symbol, protein ID, Protein Name, E-value and Identities retrieved from NBCI blastx command line tool, using UniprotKB/Swissprot database and Log2FC (positive values in bold). DEGs refers to each salinity treatment in comparison with the control NaCl, respectively, 200: 200 vs. 0 mM NaCl, 400: 400 vs 0 mM NaCl, and 600: 600 vs. 0 mM NaCl), detected by DESeq, NOISeq and edgeR analysis with a False Discovery Rate < 0.05.

Gene Symbol	Protein ID	Protein Name	E-value	Identities	Log_2FC		
					**200**	**400**	**600**
LRK10L-1.4	LRL14_ARATH	LEAF RUST 10 DISEASE-RESISTANCE LOCUS RECEPTOR-LIKE PROTEIN KINASE-like 1.4	5E-07	45	-	-3.184	-5.059
FLS2	FLS2_ARATH	LRR receptor-like serine/threonine-protein kinase FLS2	2E-63	56	-	-3.628	-4.280
LRK10	LRK10_WHEAT	Rust resistance kinase Lr10	8E-64	56	-	-	-3.262
PUB19	PUB19_ARATH	U-box domain-containing protein 19	9E-10	43	-	-	-3.031
At3g18200	WTR17_ARATH	WAT1-related protein At3g18200	1E-07	63	-	-	**2.907**
SAG101	SG101_ARATH	Senescence-associated carboxylesterase 101	8E-27	51	-	-	-3.879
UGT85A23	UGT6_CATRO	7-deoxyloganetin glucosyltransferase	7E-17	68	-	-2.807	-3.544
N	TMVRN_NICGU	TMV resistance protein N	2E-36	61	-	-	-2.898
At1g06840	Y1684_ARATH	Probable LRR receptor-like serine/threonine-protein kinase At1g06840	2E-32	68	-	-	-2.756
TIC214	TI214_HELAN	Protein TIC 214	2E-23	66	-	-	**2.907**
ZED1	ZED1_ARATH	Non-functional pseudokinase ZED1	2E-24	47	-	-	-3.222
N/A	LEC_ONOVI	Lectin	4E-08	57	-	-3.644	-5.644
RABA3	RABA3_ARATH	Ras-related protein RABA3	7E-50	85	-	-	-3.087
At3g28600	AATPA_ARATH	AAA-ATPase At3g28600	4E-48	73	-	-	-2.867
SAT	SAT_CATRO	Stemmadenine O-acetyltransferase	2E-16	47	-	**2.585**	**2.700**
DTX56	DTX56_ARATH	Protein DETOXIFICATION 56	1E-144	63	-	-	**2.929**

PK1	KPRO_MAIZE	Putative receptor protein kinase ZmPK1	2E-80	59	-	-	-3.593
CCD8	CCD8_ARATH	Carotenoid cleavage dioxygenase 8, chloroplastic	4E-05	40	-	-7.250	-6.134
DTX49	DTX49_ARATH	Protein DETOXIFICATION 49	2E-24	69	-	-	-4.755
SERK2	SERK2_ORYSJ	LRR receptor kinase SERK2	1E-03	47	-	-	-2.872
DAR4	DAR4_ARATH	Protein DA1-related 4	3E-10	47	-	-	-3.350
AHL17	AHL17_ARATH	AT-hook motif nuclear-localized protein 17	4E-10	65	-	-	-2.700
N/A	CHIT2_TULSB	Chitinase 2	5E-46	73	-	**2.267**	-
ATL55	ATL55_ARATH	E3 ubiquitin-protein ligase RING1	3E-27	83	-	-	-3.228
RPP13L4	R13L4_ARATH	Disease resistance RPP13-like protein 4	5E-35	60	-	-	-3.311
UGT85K4	UGTK4_MANES	Linamarin synthase 1	1E-130	60	-	-	-2.903
pod	PER15_IPOBA	Peroxidase 15	6E-65	64	-	-3.682	-5.530
N/A	TLP1_PRUPE	Thaumatin-like protein 1	2E-39	64	-	**3.110**	-
RBOHD	RBOHD_ARATH	Respiratory burst oxidase homolog protein D	7E-26	84	-	-	-2.555
At1g06800	PLA15_ARATH	Phospholipase A1-Igamma1, chloroplastic	9E-21	41	-	-	-4.392
WAKL9	WAKLH_ARATH	Wall-associated receptor kinase-like 9	1E-06	49	-	-	-5.044
At4g16563	ASP63_ARATH	Probable aspartyl protease At4g16563	5E-146	62	-	-	-3.370
RE1	POLR1_ARATH	Retrovirus-related Pol polyprotein from transposon RE1	3E-14	43	-	-	-3.000
At5g24080	Y5248_ARATH	G-type lectin S-receptor-like serine/threonine-protein kinase At5g24080	9E-27	57	-	-	-3.931
AOS1	AOS1_SOLLC	Allene oxide synthase 1, chloroplastic	3E-113	67	-	-	-3.774
PBL9	PBL9_ARATH	Probable serine/threonine-protein kinase PBL9	9E-08	81	-	-2.766	-3.350
At2g27310	FB119_ARATH	F-box protein At2g27310	4E-56	54	-	-	-3.829
CHS	CHSY_BETPN	Chalcone synthase	2E-178	75	-	-	-2.825
PME6	PME6_ARATH	Probable pectinesterase/pectinesterase inhibitor 6	3E-146	68	-	-5.275	-4.660
PLP1	PLP1_ARATH	Patatin-like protein 1	6E-31	92	-	-	-3.026
LBD1	LBD1_ARATH	LOB domain-containing protein 1	1E-28	92	-	-	-3.534

MAKR6	MAKR6_ARATH	Probable membrane-associated kinase regulator 6	2E-07	78	-	-	**2.861**
TAO1	TAO1_ARATH	Disease resistance protein TAO1	6E-10	41	-	-	-5.728
UGT73E1	U73E1_STERE	UDP-glycosyltransferase 73E1	1E-55	53	-	**3.524**	-
SOBIR1	SBIR1_ARATH	Leucine-rich repeat receptor-like serine/threonine/tyrosine-protein kinase SOBIR1	7E-124	80	-	-	-4.445
HSP70	HSP70_SOYBN	Heat shock 70 kDa protein	3E-70	84	-	**3.301**	**2.729**
GH3.2	GH32_ARATH	Indole-3-acetic acid-amido synthetase GH3.2	2E-118	74	-	-3.412	-3.049
SBT1.8	SBT18_ARATH	Subtilisin-like protease SBT1.8	5E-23	49	-	-3.430	-
WRKY70	WRK70_ARATH	Probable WRKY transcription factor 70	1E-29	66	-	-	-3.263
FLA12	FLA12_ARATH	Fasciclin-like arabinogalactan protein 12	5E-14	72	-	-4.542	-3.864
EXO70H1	E70H1_ARATH	Exocyst complex component EXO70H1	1E-24	54	-	-	-3.807
PUMP5	PUMP5_ARATH	Mitochondrial uncoupling protein 5	2E-24	77	-	-	-3.000
At5g10770	ASPA_ARATH	Aspartyl protease family protein At5g10770	1E-33	59	-	-	-4.170
HSP22.7	HSP41_PEA	22.7 kDa class IV heat shock protein	1E-46	52	-	-	**2.357**
ROMT	ROMT_VITVI	Trans-resveratrol di-O-methyltransferase	1E-59	50	-	-3.730	-4.829
CRT3	CALR3_ARATH	Calreticulin-3	2E-29	79	-	-	-2.942
CYP707A7	ABAH3_ORYSI	Abscisic acid 8'-hydroxylase 3	1E-13	69	-	**2.807**	-
TMK1	TMK1_ARATH	Receptor protein kinase TMK1	1E-19	76	-	**2.737**	-
OPR1	OPR1_ARATH	12-oxophytodienoate reductase 1	1E-43	72	-	-	-3.059
SDI1	SDI1_ARATH	Protein SULFUR DEFICIENCY-INDUCED 1	3E-08	56	-	**3.459**	**4.087**
At3g28050	WTR18_ARATH	WAT1-related protein At3g28050	5E-14	72	-	**2.807**	-
LECRK3	LERK3_ORYSI	G-type lectin S-receptor-like serine/threonine-protein kinase LECRK3	4E-72	56	-	-	-3.498
CYP77A3	C77A3_SOYBN	Cytochrome P450 77A3	2E-06	51	-	-	-2.831
At3g13620	PHSC_ARATH	Probable polyamine transporter At3g13620	2E-28	69	-	-3.170	-
At2g40270	Y2027_ARATH	Inactive receptor-like serine/threonine-protein kinase At2g40270	8E-06	62	-	-	-3.585
BHLH41	BH041_ARATH	Putative transcription factor bHLH041	2E-21	43	-	-	-4.858

PER64	PER64_ARATH	Peroxidase 64	5E-59	60	-	-5.977	-
COBL4	COBL4_ARATH	COBRA-like protein 4	2E-59	67	-	-4.087	-5.087
EDS1	EDS1L_ARATH	Protein EDS1L	2E-37	45	-	-	-3.426
PEPR1	PEPR1_ARATH	Leucine-rich repeat receptor-like protein kinase PEPR1	3E-27	73	**3.585**	**3.585**	**4.585**
At1g48100	PGLR4_ARATH	Polygalacturonase At1g48100	3E-89	81	-	-	**2.496**
UGT76E2	U76E2_ARATH	UDP-glycosyltransferase 76E2	8E-56	70	-	-	-2.802
YAH3	H32_LILLO	Histone H3.2	9E-42	99	-	-3.907	-
GRXC9	GRXC9_ARATH	Glutaredoxin-C9	9E-16	55	-	-	-3.104
CYP74A	CP74A_ARATH	Allene oxide synthase, chloroplastic	2E-40	78	-	-	-3.534
GATA5	GATA5_ARATH	GATA transcription factor 5	2E-42	74	-	-	-3.028
PDR1	PDR1_TOBAC	Pleiotropic drug resistance protein 1	2E-33	80	-	-	-2.572
DTX27	DTX27_ARATH	Protein DETOXIFICATION 27	3E-24	60	-	**3.763**	-
EPS1	EPS1_ARATH	Protein ENHANCED PSEUDOMONAS SUSCEPTIBILITY 1	2E-22	46	-	**3.654**	-
AVT1B	AVT1B_ARATH	Amino acid transporter AVT1B	5E-07	47	-	**2.761**	-
CCR4	ACCR4_ARATH	Serine/threonine-protein kinase-like protein CCR4	3E-83	40	-	-	-5.017
SAP12	SAP12_ARATH	Zinc finger AN1 domain-containing stress-associated protein 12	2E-26	58	-	-	-2.893
WUN1	WUN1_SOLTU	Wound-induced protein 1	1E-25	51	-	**2.829**	-
CYP707A2	ABAH2_ARATH	Abscisic acid 8'-hydroxylase 2	7E-26	92	-	**4.322**	-
NAC090	NAC90_ARATH	NAC domain-containing protein 90	2E-43	67	-	-	-3.491
N/A	ISPD_MENPI	(-)-isopiperitenol/(-)-carveol dehydrogenase, mitochondrial	3E-46	62	-	-	-3.644
Dctpp1	DCTP1_MOUSE	dCTP pyrophosphatase 1	5E-29	49	-	**2.791**	-
LECRK41	LRK41_ARATH	L-type lectin-domain containing receptor kinase IV.1	0E+00	67	-	-	-3.940
CRRSP38	CRR38_ARATH	Cysteine-rich repeat secretory protein 38	2E-08	44	-	-	-4.115
HMGB9	HMGB9_ARATH	High mobility group B protein 9	4E-13	66	-	-3.585	-
ERF112	EF112_ARATH	Ethylene-responsive transcription factor ERF112	2E-17	83	-	-	-4.658
AMT2-1	AMT21_ORYSJ	Ammonium transporter 2 member 1	3E-43	90	-	-	-3.280

N/A	POLX_TOBAC	Retrovirus-related Pol polyprotein from transposon TNT 1-94	1E-36	49	-	-	-4.459
XTH22	XTH22_ARATH	Xyloglucan endotransglucosylase/hydrolase protein 22	8E-18	61	-	-	-4.308
SOT15	SOT15_ARATH	Cytosolic sulfotransferase 15	5E-141	61	-	-3.262	-4.655
RSZ23	RZP23_ORYSJ	Serine/arginine-rich splicing factor RSZ23	1E-04	94	-	**2.202**	**2.138**
SDR1	SDR1_ARATH	(+)-neomenthol dehydrogenase	3E-18	67	-	-	**2.566**
RGA5	RGA5R_ORYSJ	Disease resistance protein RGA5	3E-04	44	-	-	-4.076
DREB1D	DRE1D_ARATH	Dehydration-responsive element-binding protein 1D	2E-32	71	-	-	-4.728
At2g30020	P2C25_ARATH	Probable protein phosphatase 2C 25	8E-32	74	-	-	-3.983
MYBAS1	MYBA1_ORYSJ	Myb-related protein MYBAS1	3E-09	74	-	**4.129**	-
CBP60B	CB60B_ARATH	Calmodulin-binding protein 60 B	3E-04	67	-	-	-4.087
CRK10	CRK10_ARATH	Cysteine-rich receptor-like protein kinase 10	1E-61	47	-	-	-5.000
At1g11050	Y1105_ARATH	Probable receptor-like protein kinase At1g11050	1E-45	81	-	-	-4.000
PRB1	PRB1_ARATH	Pathogenesis-related protein 1	6E-66	62	-	-	-4.298
GAE6	GAE6_ARATH	UDP-glucuronate 4-epimerase 6	6E-97	93	-	-2.855	-
PUB17	PUB17_ARATH	U-box domain-containing protein 17	7E-35	70	-	-	-3.233
AMT3-3	AMT33_ORYSJ	Ammonium transporter 3 member 3	6E-24	73	-	-3.322	-
N/A	CRJ35_CRYJA	Pathogenesis-related thaumatin-like protein 3.5	9E-08	79	-	**3.954**	-
RL5	RADL5_ARATH	Protein RADIALIS-like 5	2E-28	70	-	-7.794	-7.794
GNS1	E13B_PRUPE	Glucan endo-1,3-beta-glucosidase, basic isoform	1E-157	68	-	-	-3.321
SHT	SHT_ARATH	Spermidine hydroxycinnamoyl transferase	9E-28	47	-	-3.700	-
N/A	C7A12_PANGI	Cytochrome P450 CYP736A12	1E-70	63	-	-3.154	-3.463
CXE6	CXE6_ARATH	Probable carboxylesterase 6	8E-14	40	-	-	-4.139
At4g03230	Y4230_ARATH	G-type lectin S-receptor-like serine/threonine-protein kinase At4g03230	1E-46	70	-	-	-3.066
RL3	RADL3_ARATH	Protein RADIALIS-like 3	1E-28	75	-	-4.262	-3.399
GLR2.8	GLR28_ARATH	Glutamate receptor 2.8	4E-05	69	-3.248	-5.248	-5.248

CYP78A5	C78A5_ARATH	Cytochrome P450 78A5	6E-19	61	**2.893**	-	-
PLP2	PLP2_ARATH	Patatin-like protein 2	1E-44	66	-	-	-4.209
LAC22	LAC22_ORYSJ	Laccase-22	4E-138	64	-	-	-5.183
At4g14490	Y4449_ARATH	FHA domain-containing protein At4g14490	1E-25	53	-	**2.096**	-
SEOA	SEOA_ARATH	Protein SIEVE ELEMENT OCCLUSION A	8E-21	49	-	-	-3.524
GDH2	DHE2_ARATH	Glutamate dehydrogenase 2	7E-43	93	**3.322**	**5.087**	**3.807**
CAO	CAO_ORYSJ	Chlorophyllide a oxygenase, chloroplastic	2E-08	100	-	-	**4.248**
AATP1	ASD_ARATH	AAA-ATPase ASD, mitochondrial	3E-15	50	-	-	-4.807
LECRK91	LRK91_ARATH	L-type lectin-domain containing receptor kinase IX.1	4E-33	68	-	-3.503	-5.087
SIT1	SIT1_ORYSJ	L-type lectin-domain containing receptor kinase SIT1	9E-21	55	-	-	-3.051
WAKL6	WAKLF_ARATH	Wall-associated receptor kinase-like 6	1E-07	53	-	-3.524	-
IRX15-L	IX15L_ARATH	Protein IRX15-LIKE	3E-25	61	-	-2.974	-3.459
HMG2	HMDH2_GOSHI	3-hydroxy-3-methylglutaryl-coenzyme A reductase 2	2E-41	64	-	-	-2.749
CML37	CML37_ARATH	Calcium-binding protein CML37	1E-19	50	-	-	-3.600
SERK4	SERK4_ARATH	Somatic embryogenesis receptor kinase 4	1E-05	40	-	-	-3.970
N/A	LECS_VATMA	Seed lectin	2E-16	40	-	-	-4.700
At1g30200	FB19_ARATH	F-box protein At1g30200	7E-06	40	-	-	-3.152
UCH2	UCH2_ARATH	Ubiquitin carboxyl-terminal hydrolase 2	1E-09	93	-	-	-3.645
PHT1-12	PT112_ORYSJ	Probable inorganic phosphate transporter 1-12	2E-32	79	-	-	-3.577
